# The Consumption of Opium, in China

**Published:** 1889-05

**Authors:** 


### THE CONSUMPTION OF OPIUM IN CHINA.

A Chinese Customs yellow-book contains some valuable and interesting information on the growth and consumption of opium in China. The growth of native opium has assumed large proportions during recent years. Every province produces it, the only large areas where it is not grown being the islands of Formosa and Hainan. The opium is chiefly consumed locally, only a comparatively small percentage going out of the district or province where it is grown. The quality of the native article has much improved, and this fact, combined with the increased growth, has led to a great reduction in the import of foreign opium, as the falling-off in the Indian revenue testifies. Even at the treaty ports native opium is used very largely. It is preferred in many cases, because it can be smoked seven or eight times, while the foreign drug can be smoked three times at the most. The native is often mixed with the foreign, but smokers object to this being done, because it spoils the ashes, which command a price. Economy comes into the question of preference, for only the wealthiest consume the foreign article. A great deal of smuggling goes on in order to evade the tax. In the far West where cash and silver are scarce, opium takes the place of money in many localities as the basis of barter. When starting on a journey, a native carries his estimated expenses in the form of opium, selling here and there just as much as he requires. Even students going to Pekin, carry their funds in the form of opium.

